# scSurv: a deep generative model for single-cell survival analysis

**DOI:** 10.1093/bioinformatics/btaf671

**Published:** 2025-12-22

**Authors:** Chikara Mizukoshi, Yasuhiro Kojima, Shuto Hayashi, Ko Abe, Daisuke Kasugai, Teppei Shimamura

**Affiliations:** Department of Computational and Systems Biology, Division of Biological Data Science, Medical Research Laboratory, Institute for Integrated Research, Institute of Science Tokyo, Tokyo 113-8510, Japan; Division of Systems Biology, Graduate School of Medicine, Nagoya University, Nagoya, Aichi 466-8550, Japan; Nagoya University Hospital, Nagoya, Aichi 466-8560, Japan; Laboratory of Computational Life Science, National Cancer Center Research Institute, Tokyo, 104-0045, Japan; Department of Computational and Systems Biology, Division of Biological Data Science, Medical Research Laboratory, Institute for Integrated Research, Institute of Science Tokyo, Tokyo 113-8510, Japan; Department of Computational and Systems Biology, Division of Biological Data Science, Medical Research Laboratory, Institute for Integrated Research, Institute of Science Tokyo, Tokyo 113-8510, Japan; Department of Emergency and Critical Care Medicine, Graduate School of Medicine, Nagoya University, Nagoya, Aichi 466-8550, Japan; Institute of Nano-Life-Systems, Institutes of Innovation for Future Society, Nagoya University, Nagoya, Aichi 464-8601, Japan; Department of Computational and Systems Biology, Division of Biological Data Science, Medical Research Laboratory, Institute for Integrated Research, Institute of Science Tokyo, Tokyo 113-8510, Japan; Division of Systems Biology, Graduate School of Medicine, Nagoya University, Nagoya, Aichi 466-8550, Japan

## Abstract

**Motivation:**

Single-cell omics analysis has unveiled the heterogeneity of various cell types within tumors. However, no methodology currently reveals how this heterogeneity influences cancer patient survival at single-cell resolution. Here, we introduce scSurv, combining a Cox proportional hazards model with a deep generative model of single-cell transcriptome, to estimate individual cellular contributions to clinical outcomes.

**Results:**

The accuracy of scSurv was validated using both simulated and real datasets. This method identifies cells associated with favorable or adverse prognoses and extracts genes correlated with their contribution levels. In melanoma, scSurv reproduces known prognostic macrophage classifications and facilitates hazard mapping through spatial transcriptomics in renal cell carcinoma. We also identified genes consistently associated with prognosis across multiple cancers and demonstrated the applicability of this method to infectious diseases. scSurv is a novel framework for quantifying the heterogeneity of individual cellular effects on clinical outcomes.

**Availability:**

The implementation of scSurv is available on GitHub (https://github.com/3254c/scSurv) and Zenodo (https://doi.org/10.5281/zenodo.17793054).

## Introduction

Survival analysis is a statistical method widely used across various fields to model the time until a specific event occurs, accounting for the influence of multiple covariates. These models typically consist of two primary components: the baseline hazard function, which represents the underlying hazard when all covariates are absent, and the effect parameters, which quantify how explanatory covariates influence the hazard function. Among them, the Cox proportional hazards model is the most widely used. This semi-parametric model assumes covariates have a multiplicative effect on the hazard function and allows the estimation of effect parameters without specifying the form of the baseline hazard function.

The Cox proportional hazards model has significantly enhanced the analysis of associations between molecular profiles-obtained through next-generation sequencing (NGS) and mass spectrometry-and survival times, enabling the identification of prognostic factors and the development of predictive models (Gentles *et al.* 2015, Zhang *et al.* 2016, Thorsson *et al.* 2018). However, traditional approaches based on population averages are limited, particularly when addressing the cellular heterogeneity inherent in diseases and the specific role of various tissues and cell types in pathology. The diversity of cells and intercellular interactions within tumors and the tumor microenvironment influences disease progression and treatment responses (Chen and Mellman 2017, Dagogo-Jack and Shaw 2018, Rad *et al.* 2020), underscoring the need for more precise analyses at the cellular level.

Recent advances in single-cell sequencing technologies have enabled the high-resolution characterization of gene expression patterns and individual cell states, offering novel insights into disease mechanisms and identifying therapeutic targets (Regev *et al.* 2017, Papalexi and Satija 2018, Svensson *et al.* 2018). Nevertheless, large-scale cohorts integrating single-cell data with clinical information remain limited due to technical and economic constraints. In contrast, bulk RNA sequencing data linked to clinical outcomes are widely available. Despite their lower resolution, these data are valuable for analyzing relationships between molecular profiles and clinical outcomes.

Recent computational advancements have enabled the estimation of cell type proportions from bulk RNA sequencing data (Newman *et al.* 2019, Wang *et al.* 2019b, Chu *et al.* 2022). However, deconvolution models based on cell types inherently restrict analyses to this level, failing to capture variations in individual cell states or functions. These limitations hinder our ability to elucidate detailed biological mechanisms, including subtle shifts in cell states or associations between specific subtypes and survival outcomes. Consequently, critical biological insights may be overlooked, particularly in complex diseases like cancer, which exhibit pronounced intratumoral heterogeneity.

We addressed these limitations by developing scSurv, a novel method for single-cell survival analysis that extends the Cox proportional hazards model to incorporate cellular heterogeneity. Using single-cell RNA sequencing data as a reference, scSurv deconvolves bulk RNA-seq data to infer single-cell proportions and quantifies their individual contributions to patient outcomes. scSurv systematically identifies cells contributing to disease risk and their specific gene signatures by analyzing the association between survival time and cell abundance.

Simulations demonstrated that scSurv accurately predicts patient prognosis using single-cell proportions derived from deconvolution. Applying this method to The Cancer Genome Atlas (TCGA) data, we found that it could predict survival time of patients excluded from training across multiple cancers. scSurv identified specific cells influencing patient prognosis and the genes associated with these outcomes in melanoma. It also successfully reproduced known macrophage classifications affecting patient prognosis. Spatial transcriptomic analysis of renal cell carcinoma enabled tissue-wide hazard mapping and identified distinct prognosis-associated spatial regions. Furthermore, we identified genes contributing to prognosis across multiple cancers. Finally, applying this method to infectious diseases demonstrated its utility beyond cancer and survival outcomes. This novel approach elucidates the contributions of individual cells to clinical outcomes and offers new perspectives in clinical analyses.

## Materials and methods

### Concept of scSurv

We developed a novel method called scSurv, a deep generative model for single-cell survival analysis, to facilitate survival analysis at the single-cell level and to uncover biological mechanisms in diseases. scSurv involves the following steps: (1) This method uses single-cell RNA sequencing (scRNA-seq) data as a reference, following our previous framework (Kojima *et al.* 2024). Bulk RNA sequencing (bulk RNA-seq) data are deconvoluted at the cellular level using latent cell states obtained from a variational autoencoder (VAE). (2) scSurv estimates the hazard function using a Cox proportional hazards model, extended by combining the estimated proportion of each single cell within the bulk samples and the regression coefficients obtained from the latent cell state. These regression coefficients are interpreted as the contributions of individual cells to clinical outcomes. This model enables the evaluation of hazard contributions at the single-cell level and enhances their consistency among cells with similar cell states.

The trained model offers three main analyses: (1) Quantification of individual cells’ contributions to clinical outcomes. (2) Identification of prognosis-associated gene sets. (3) Mapping of spatial hazard distributions using spatial transcriptome data.

Through these applications, scSurv provides a comprehensive and interpretable framework that reveals heterogeneity in the clinical significance of cells. This framework enables the identification of novel cell populations and genes involved in the prognosis. This method is available as an open-source Python package on GitHub (https://github.com/3254c/scSurv).

### scSurv framework

The scSurv framework consists of three main steps: learning latent states through VAE, deconvolution of bulk data, and estimation of regression coefficients in hazard functions for each cell using the extended Cox proportional hazards model. These steps are performed sequentially. The parameters learned in each step are then frozen and are not updated in the subsequent steps. The first two steps are similar to those in our previous study, DeepCOLOR (Kojima *et al.* 2024). scSurv employs a conditional VAE framework (Kingma *et al.* 2014) similar to scVI (Lopez *et al.* 2018) to handle scRNA-seq data collected from multiple patients as input. For all training steps, 85% of cells were randomly assigned to the training set, 10% to the validation set, and 5% to the test set. This same split was used for the VAE, deconvolution, and extended Cox steps. For the extended Cox step, bulk RNA-seq data were split into 60% training, 20% validation, and 20% test sets. The validation set is used for early stopping in order to suppress overfitting. The test set is used to compute reconstruction accuracy and the c-index with the trained model and to provide the final evaluation of model accuracy. The VAE compresses raw gene expression into low-dimensional latent cell representations, which can be treated as summaries of essential cellular information. scSurv utilizes the loss function of the Cox proportional hazards model to learn the contribution to the hazard function using the proportion of each single cell in each bulk sample as covariates. The proportions of the cells and their contributions to the hazard function learned in this process depends on the latent states of the cells. Consequently, cells with similar latent states are estimated to have similar contributions. This approach enables the model to effectively learn the contributions of 10,000 cells. A summary of the architecture, optimization settings, and hyperparameters is available in the supplementary materials (Table S1). These materials also include a detailed description of the model training process.

### Derivation of the stochastic latent representation of the single-cell transcriptome

We define a probabilistic model for raw counts of single-cell transcriptomes. Let $z_{c}\in R^{M}$ represent the low-dimensional latent cell state, where *M* is the dimension of the latent space and the subscript *c* denotes each cell. We assume a Gaussian prior distribution for $z_{c}$:


$$p(z_{c})=N(z_{c}|0,I)$$

Let *G* be the number of genes and $x_{c}\in R^{G}$ be the raw counts of the single-cell transcriptome. We assume that given $z_{c}$, the counts follow a Poisson distribution:


$$\begin{array}{c} p(x_{cg}|z_{c},p_{c})=\text{Poisson}(x_{cg}|{\hat{x}}_{cg})\\ {\hat{x}}_{c}=l_{c}f_{\theta }(z_{c},p_{c}) \end{array}$$

Here, $l_{c}\in R$ is the mean of all genes for each cell, $p_{c}\in {\left[0,1\right]}^{P}$ represents the batch information (patient information in this context), *P* is the total number of batches, and $p_{c,k}=1$ indicates that cell *c* is collected from patient *k*. The function $f_{\theta }(z_{c},p_{c})\in R^{G}$ is the decoding neural network of the latent cell state.

To obtain the latent cell state $z_{c}$, we use a VAE framework to represent the posterior distribution $q(z_{c}|x_{c},p_{c})$. We assume that the posterior distribution follows a Gaussian distribution:


$$q(z_{c}|x_{c},p_{c})=N(z_{c}|\mu_{\Phi }(x_{c},p_{c}),\text{diag}(\sigma_{\Phi }(x_{c},p_{c})))$$

Where $\mu_{\Phi }(x_{c})$ and $\sigma_{\Phi }(x_{c})$ denote the encoding neural networks.

We maximize the following Evidence Lower Bound to optimize the parameters of the generative model and variational distribution:


$$L(\theta ,\phi )=\sum_{c=1}^{C}\left(\text{log}\;p(x_{c}|z_{c}^{\prime },p_{c})-D_{KL}(q(z_{c}|x_{c},p_{c})||p(z_{c}))\right)$$

Where $z_{c}^{\prime }$ is obtained from $q(z_{c}|x_{c},p_{c})$ using the reparameterization trick.

### Probabilistic model of bulk transcriptome data and spatial transcriptome data

We model the expression $e_{b}\in R^{G}$ of bulk *b* by following a negative binomial distribution:


$$\begin{array}{c} p(e_{bg}|\mu_{bg}^{\mathrm{bulk}},\alpha_{g}^{\mathrm{bulk}})=\text{NegativeBinomial}(e_{bg}|\mu_{bg}^{\mathrm{bulk}},\alpha_{g}^{\mathrm{bulk}})\\ \mu_{bg}^{\mathrm{bulk}}=r_{g}^{\mathrm{bulk}}\sum_{c=1}^{C}f_{\theta }{\left(z_{c},p_{c}\right)}_{g}m_{\theta^{\mathrm{bulk}}}{\left(z_{c}\right)}_{b}+o_{g}^{\mathrm{bulk}} \end{array}$$

Where $\mu_{bg}^{\mathrm{bulk}}\in R$ is the mean parameter, $\alpha_{g}^{\mathrm{bulk}}\in R$ is the dispersion parameter, $r_{g}^{\mathrm{bulk}}\in R$ represents the capture rate of each gene in bulk RNA-seq compared to scRNA-seq, and $o_{g}^{\mathrm{bulk}}\in R$ is the shift parameter for each gene. Both $r_{g}^{\mathrm{bulk}}$ and $o_{g}^{\mathrm{bulk}}$ are constrained to be positive scalar parameters shared across all bulk samples, ensuring model identifiability. $m_{\theta^{\mathrm{bulk}}}(z_{c})\in R^{B}$ is a neural network that outputs the proportion of each cell in each bulk sample given the latent cell state as the input, satisfying:


$$\sum_{c=1}^{C}m_{\theta^{\mathrm{bulk}}}{\left(z_{c}\right)}_{b}=1$$

We optimize the parameters $\theta^{\mathrm{bulk}}$, $r^{\mathrm{bulk}}$, $o^{\mathrm{bulk}}$, $\alpha^{\mathrm{bulk}}$ by maximizing the following log-likelihood:


$$L(\theta^{\mathrm{bulk}},r^{\mathrm{bulk}},o^{\mathrm{bulk}},\alpha^{\mathrm{bulk}})=\sum_{b=1}^{B}\;\text{log}\;p(e_{b}|\mu^{\mathrm{bulk}},\alpha^{\mathrm{bulk}})$$

Similarly, we model the expression $e_{s}\in R^{G}$ of Visium spot *s* following a negative binomial distribution:


$$\begin{array}{c} p(e_{sg}|\mu_{sg}^{\mathrm{spot}},\alpha_{g}^{\mathrm{spot}})=\text{NegativeBinomial}(e_{sg}|\mu_{sg}^{\mathrm{spot}},\alpha_{g}^{\mathrm{spot}})\\ \mu_{sg}^{\mathrm{spot}}=r_{g}^{\mathrm{spot}}\sum_{c=1}^{C}f_{\theta }{\left(z_{c},p_{c}\right)}_{g}m_{\theta^{\mathrm{spot}}}{\left(z_{c}\right)}_{s}+o_{g}^{\mathrm{spot}} \end{array}$$

Where the parameters are defined analogously to the bulk model, with $m_{\theta^{\mathrm{spot}}}(z_{c})\in R^{S}$, where *S* denotes the number of spots, satisfying:


$$\sum_{c=1}^{C}m_{\theta^{\mathrm{spot}}}{\left(z_{c}\right)}_{s}=1$$

We optimize the parameters $\theta^{\mathrm{spot}}$, $r^{\mathrm{spot}}$, $o^{\mathrm{spot}}$, $\alpha^{\mathrm{spot}}$ by maximizing the following log-likelihood:


$$L(\theta^{\mathrm{spot}},r^{\mathrm{spot}},o^{\mathrm{spot}},\alpha^{\mathrm{spot}})=\sum_{s=1}^{S}\;\text{log}\;p(e_{s}|\mu^{\mathrm{spot}},\alpha^{\mathrm{spot}})$$

### Single-cell cox proportional hazards model

We define the hazard function for each bulk sample as:


$$\begin{array}{c} h{\left(t\right)}_{b}=h_{0}(t)\;\text{exp}(\eta_{b})\\ \eta_{b}=\sum_{c=1}^{C}\beta_{\theta^{\prime }}(z_{c})m_{\theta^{\mathrm{bulk}}}{\left(z_{c}\right)}_{b} \end{array}$$

Where $\beta_{\theta^{\prime }}(z_{c})\in R^{c}$ is a neural network that outputs regression coefficients given latent cell states as input, and $h_{0}(t)$ is the baseline hazard function, which remains unspecified.

Following the Cox proportional hazards model, we optimize the parameters $\theta^{\prime }$ of $\beta_{\theta^{\prime }}(z_{c})$ by maximizing the partial log-likelihood. Using the Breslow method (Breslow 1974), we maximize:


$$L_{\mathrm{breslow}}(\theta^{\prime })=\sum_{b:I_{b}=1}\left(\eta_{b}-\text{log}\;\sum_{i:T_{i}\geq T_{b}}\;\text{exp}\;(\eta_{i})\right)$$

Where $I_{b}=1$ indicates the occurrence of an event (death) for the patient of bulk *b*. $T_{b}$ represents the time of death when $I_{b}=1$, or the last contact time when $I_{b}=0$.

Using the Efron method (Efron 1977), we maximize:


$$\begin{array}{c} L_{\mathrm{efron}}(\theta^{\prime })=\sum_{b:I_{b}=1}\left\{\eta_{b}-\text{log}\;\left(\sum_{i:T_{i}\geq T_{b}}\text{exp}(\eta_{i})-\frac{r_{b}-1}{d_{b}}\sum_{l:T_{l}=T_{b},E_{l}=1}\text{exp}(\eta_{l})\right)\right\} \end{array}$$

Where $d_{b}$ is the total number of patients experiencing events simultaneously at $T_{b}$, and $r_{b}$ is the 1-based index indicating the position of bulk *b* in tied data, with $r_{b}=1$ when $d_{b}=1$.

## Results

### Validation using simulated datasets

We validated the performance of scSurv using simulated datasets. Whereas the existing bulk RNA-seq deconvolution methods only perform cluster-level resolution, scSurv performs deconvolution at the single-cell level. Through simulations, we assessed the accuracy of scSurv’s deconvolution and the precision of estimating the contributions to the hazard function based on the inferred cell proportions. Our results showed that under realistic conditions, single-cell level deconvolution achieves higher accuracy and is more effective for hazard regression than cluster-level methods.

We first clustered scRNA-seq data and assigned cluster labels to generate the simulated data. We then split the data into two subsets: one used as a reference and the other used to create pseudo-bulk samples. We randomly determined the proportion of each cell cluster within the bulk samples and generated pseudo-bulk RNA-seq data by aggregating their expression. We assigned regression coefficients in the hazard function to each cluster and set the survival times for each pseudo-bulk sample based on the Cox proportional hazards model.

We evaluated scSurv’s performance in deconvoluting bulk data and compared it with existing methods such as CIBERSORTx (Newman *et al.* 2019), MuSiC (Wang *et al.* 2019b), and BayesPrism (Chu *et al.* 2022), which perform cluster-level deconvolution of bulk RNA-seq using scRNA-seq data as a reference. However, these methods do not achieve single-cell resolution. In realistic settings, the boundaries of meaningful cell populations may not align with the provided cluster labels. Therefore, we evaluated two scenarios: one in which we provided the same cluster labels used to generate the pseudo-bulk data for these methods and the other in which we provided labels with different boundaries.

**Figure 1.**
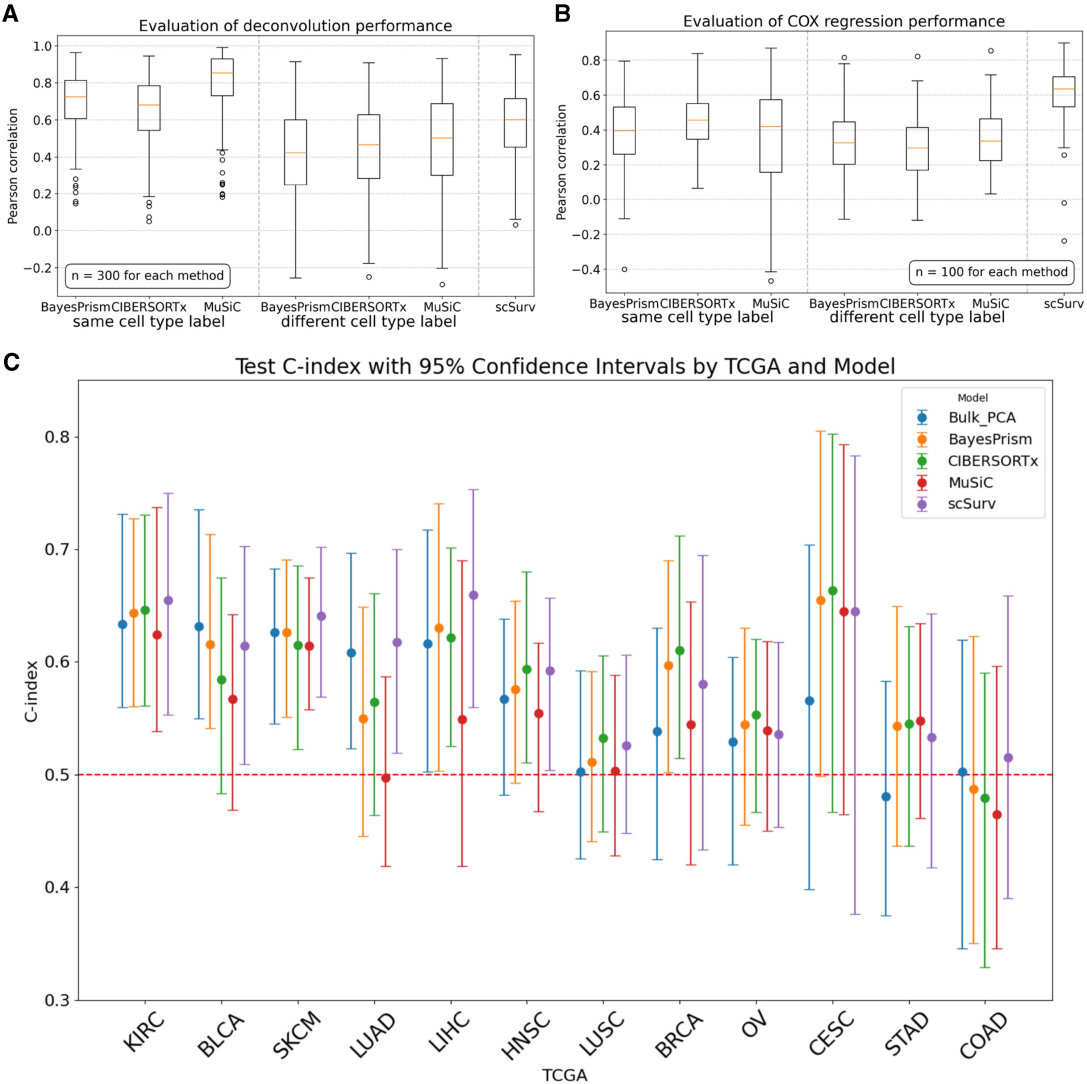
Evaluation of scSurv. (**A**) Deconvolution accuracy of scSurv, BayesPrism, CIBERSORTx, and MuSiC using 300 simulated pseudo-bulk samples. Pearson correlation between estimated proportions and ground truth values was calculated. BayesPrism, CIBERSORTx, and MuSiC were evaluated under two conditions: using same or different cluster labels between pseudo-bulk generation and deconvolution. (**B**) Accuracy of regression coefficient estimation using simulated data across methods. Pearson correlation between estimated and ground truth regression coefficients was calculated across 100 datasets, which were generated using the same 300 pseudo-bulk samples with different survival time settings by varying coefficient values. For BayesPrism, CIBERSORTx, and MuSiC, coefficients were estimated using the lifelines library based on deconvoluted cell type proportions under two conditions. (**C**) The performance of scSurv, the combination of the existing methods for bulk deconvolution with the Cox proportional hazards model, and the combination of bulk PCA with the hazard model were evaluated across 12 TCGA cancer types using the c-index. 95% confidence intervals were calculated from 100 iterations with different train/validation/test splits. scSurv achieved a test c-index > 0.5 in KIRC, BLCA, SKCM, LUAD, LIHC and HNSC. Lower performance in certain cancers suggests insufficient prognostic information in bulkRNA-seq data.

The cell-type proportions estimated by scSurv were positively correlated with the ground-truth values in the simulation (Fig. 1A). When the existing methods were given the same cluster labels used to generate pseudo-bulk RNA-seq data, their estimated cluster-level proportions were more accurate than those of scSurv. However, when different labels were provided, scSurv’s deconvolution accuracy was higher than the existing methods. This finding indicates that cluster-level deconvolution methods are limited by performance variability depending on the cluster labels provided and the clustering process. In contrast, scSurv does not use cell type annotations, so its performance is unaffected by the introduced noise. scSurv’s single-cell level deconvolution avoids the bias introduced by clustering and produces consistent results.

Additionally, when estimating the contribution of each cluster to the hazard function using the inferred cell proportions, scSurv’s estimates were positively correlated with the ground-truth values (Fig. 1B). In both cases, where the same cluster labels were used as those in generating the pseudo-bulk RNA-seq and where different labels were used, scSurv’s estimation accuracy for contributions was higher than that of the existing methods. We found that cluster-level deconvolution methods produce results that vary depending on the clusters provided and fail to accurately estimate contributions when the cluster labels used in the hazard estimation differed from those used to determine hazard values in the simulation. These results validate the accuracy of scSurv’s estimations and support the advantage of performing single-cell level estimation of the contribution to the hazard function.

### Evaluating generalization performance using TCGA datasets

We applied scSurv to real datasets from TCGA. We selected 12 cancer types that had bulk RNA-seq data from over 300 patients to ensure learning stability and reported the c-index and Integrated Brier Score (IBS) (Fig. 1C, Supplementary Fig. S1E–S1F). In the training data, scSurv successfully predicted the hazard function based on cell proportions (Supplementary Fig. S1D). In the test data, scSurv demonstrated generalization performance by achieving a 95% confidence interval for the concordance index above 0.5 in multiple cancers (Fig. 1C). However, in some cancer types, the 95% confidence interval for estimation accuracy crossed 0.5, indicating difficulty in prediction. In these cancers, the confidence intervals of the c-index for the hazard estimation also crossed 0.5, even when we directly used the bulk RNA-seq data for hazard estimation, suggesting that bulk RNA-seq data may lack sufficient information to accurately predict the hazard function. This observation is consistent with previous studies that reported challenges in predicting prognosis using TCGA bulk RNA-seq data for certain cancer types (Cheerla and Gevaert 2019, Huang *et al.* 2020). Moreover, for the cancers with high estimation accuracy using bulk RNA-seq data (the lower bound of 95% CI > 0.5), scSurv’s estimation accuracy outperformed the existing methods, except for BLCA. For the cancers with low prediction accuracy (the lower bound of 95% CI < 0.5), we found that the 95% CI of the c-index crossed 0.5 for most of the methods, suggesting that meaningful estimation of the hazard and their comparison were difficult in these cancers. From these findings, scSurv achieved accurate estimations across several cancer types, indicating that single-cell wise estimation of the hazard contribution by scSurv leads to more accurate prognosis prediction from bulk transcriptome data.

### Identifying cells and genes associated with melanoma prognosis

**Figure 2.**
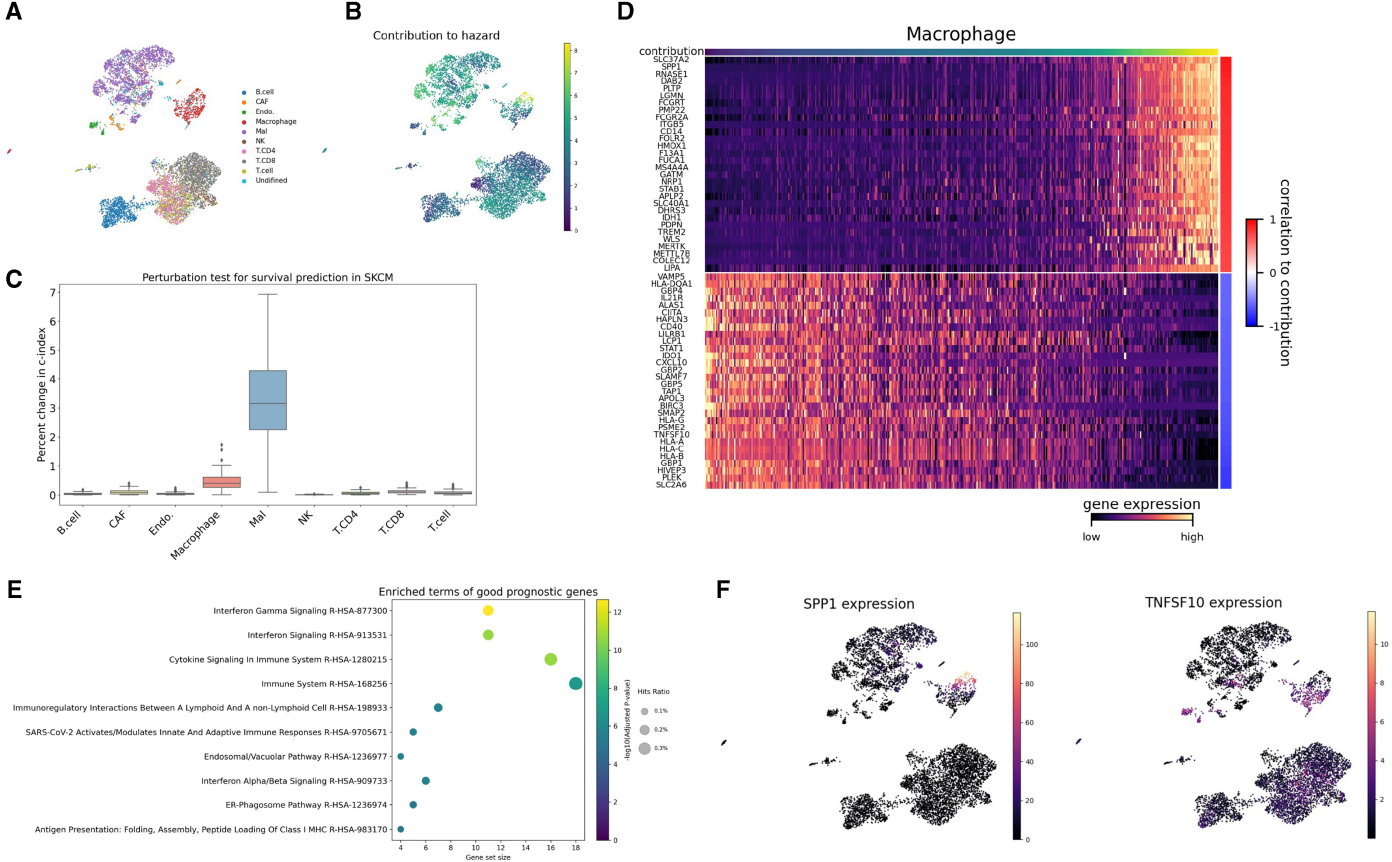
scSurv reveals prognostic cells and genes in melanoma. (**A**) UMAP visualization of cell type annotations in melanoma dataset. (**B**) Single-cell contributions estimated by scSurv. Single-cell contributions are regression coefficients in the hazard function, which are estimated using single-cell proportions; both the contributions and proportions are derived from the latent states of a VAE. Higher values indicate adverse prognostic impact. (**C**) Permutation test identifying prognostically important clusters. Estimated hazard contributions were permuted across cells within each cluster, and the resulting decrement in the c-index was quantified to assess the impact of each cluster on hazard estimation. (**D**) Heatmap showing expression of genes correlated with estimated contributions. The expression reconstructed from the latent variables was used. Top 30 positively and negatively correlated genes are displayed. The estimated single-cell contributions are shown in the upper panel. (**E**) Dot plot showing gene set enrichment analysis results using the top 30 negatively correlated genes with contributions. Dot size represents the proportion of enriched genes, the color indicates statistical significance, and the x-axis shows the number of genes in each pathway. (**F**) UMAP visualization of reconstructed SPP1 and TNFSF10 expression, showing concordance with their prognostic contributions.

We applied scSurv to a melanoma cohort (TCGA-SKCM). Melanoma is the most lethal type of skin cancers worldwide.(Leonardi *et al.* 2018) Our analysis revealed that specific populations of cancer cells, fibroblasts, endothelial cells, and macrophages adversely affected survival outcomes (Fig. 2A and B). We permuted the estimated contributions across cells within each cell cluster and quantified the decrement in the c-index to determine which cell clusters are important for prognostic prediction (Fig. 2C). Here, we found that cancer cells and macrophages exhibited a larger decrement, indicating that the heterogeneity of hazard contributions within these cell types had larger effects on the prognosis than the other cell types. Although intratumoral and intertumoral heterogeneity of cancer cells are documented factors influencing patient outcomes (Grzywa *et al.* 2017), recent findings additionally highlight the critical role of tumor-associated macrophages in shaping the tumor microenvironment and affecting patient prognosis (DeNardo and Ruffell 2019). Accordingly, our analysis focused on macrophages.

We isolated macrophages and identified genes whose expression correlated with scSurv estimated contributions (Fig. 2D). Gene set enrichment analysis revealed that interferon gamma signaling pathways were associated with favorable prognosis (Fig. 2E). This finding is consistent with those of previous studies (Wang *et al.* 2017). Additionally, the SPP1 gene characterizes macrophage subsets that contribute to tumor promotion (Bill *et al.* 2023). In contrast, the TNFSF10 gene encodes TNF-related apoptosis-inducing ligand (TRAIL), which promotes antitumor activity by inducing tumor cell apoptosis (Eisinger *et al.* 2020; Gunalp *et al.* 2023). In scSurv’s estimations, SPP1 expression positively correlated with adverse prognosis, whereas TNFSF10 expression correlated negatively (Fig. 2F). These results indicate that scSurv can classify macrophages into prognostic subsets, aligning with previous studies.

Our results confirm scSurv effectiveness in identifying prognostically relevant cells and genes. The consistency of these findings with existing knowledge highlights scSurv’s utility in biological analyses.

### Integration with spatial transcriptomics data in renal cell carcinoma

**Figure 3.**
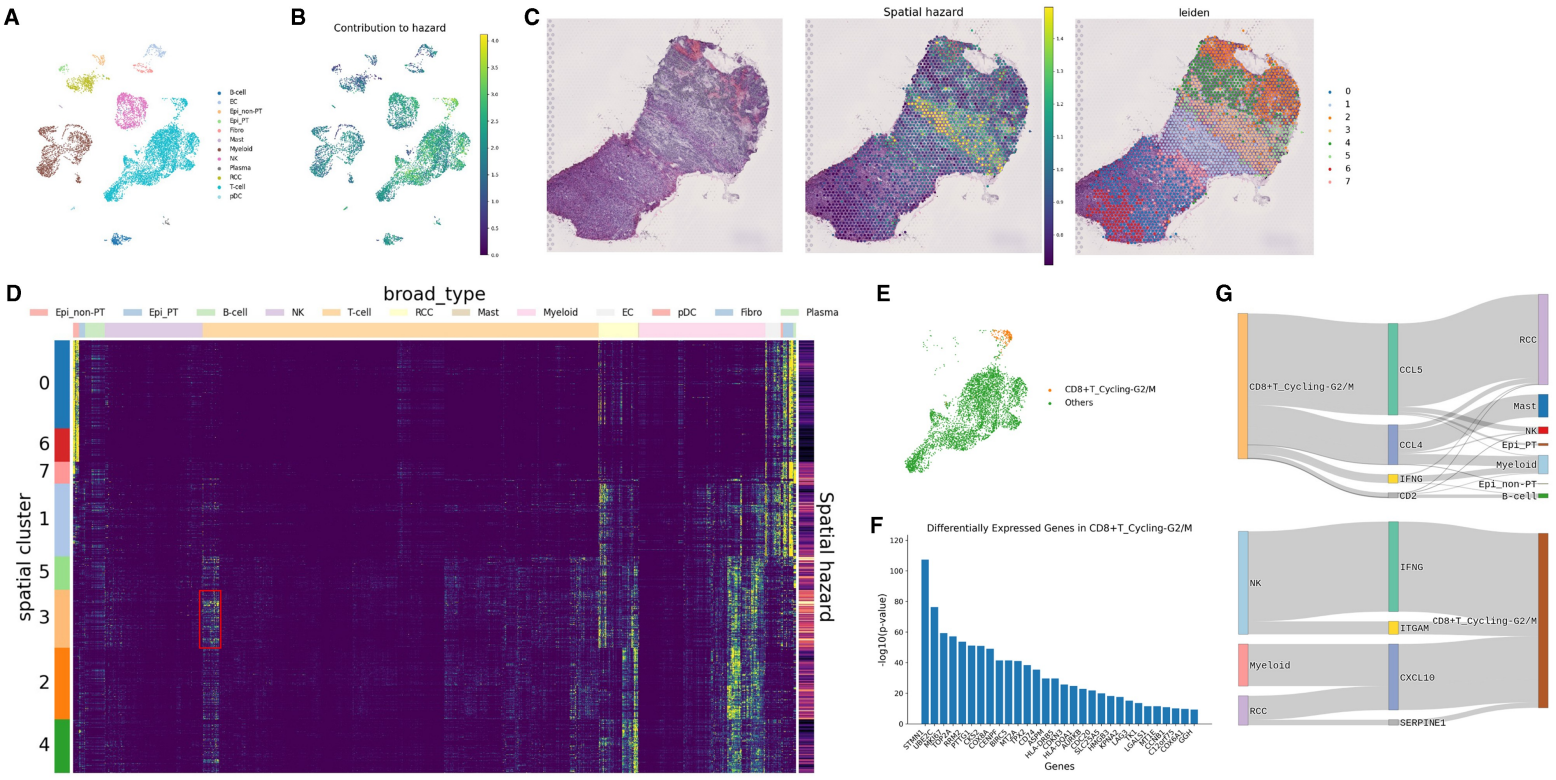
Integrated analysis using spatial transcriptomics in renal cell carcinoma. (**A**) UMAP visualization of cell type annotations in renal cell carcinoma dataset. (**B**) Single-cell contributions estimated by scSurv. Higher values indicate adverse prognostic impact. (**C**) Spatial visualization showing H&E image, mapped spatial hazards, and spot-level clustering. The minimum or maximum hazard value of the top 2.5% was forced to the 2.5% and 97.5% quantile values to prevent extreme values from affecting the visualization. (**D**) Heatmap of single-cell contributions adjusted for cell proportions. Cluster 3 showed the highest spatial hazard, with specific T cell populations. (**E**) UMAP visualization of cluster 3 specific T cell populations. (**F**) Differentially expressed genes in identified T cell clusters. (**G**) Sankey plot showing cell cell communications between the identified T cell cluster and other cell types predicted by NicheNet.

We expanded the scSurv analysis by integrating spatial transcriptomics data. First, we conducted the standard scSurv estimation to assign contributions to each cell using a renal cell carcinoma cohort (TCGA-KIRC) and scRNA-seq data (Li *et al.* 2022b) (Fig. 3A and B). Next, we estimated the cellular composition for each spot in the spatial transcriptome. We assigned a spatial hazard score to each spot based on single-cell proportions and individual cell contributions (Fig. 3C). Regions with high spatial hazards were distributed between areas containing cancer cells and those containing normal cells (Supplementary Fig. S2A).

We clustered the spots based on their expressions to obtain spatial clusters, calculated the average hazard for each spatial cluster, and focused on clusters with particularly high hazards (Fig. 3D). Notably, we found that proliferative CD8-positive T cells specifically influenced the hazard function within this cluster (Fig. 3E, Supplementary Fig. S2B–S2D). This population of T cells is characterized by the expression of the Ki-67 gene (Fig. 3F) and is associated with poor prognosis in renal cell carcinoma (Blessin *et al.* 2021). Analysis of intercellular communication networks involving spatially co-localized cells in this population identified several prognostically relevant genes, including CCL4, CCL5, IFNG, ITGAM, and CXCL10 (Wang *et al.* 2019a, Zhang *et al.* 2021, Xu *et al.* 2022, Qu *et al.* 2022, Perelli *et al.* 2023) (Fig. 3G). Integrating scSurv with spatial transcriptomics allowed us to perform hazard mapping across tissue sections, identify specific areas linked to patient prognosis, and examine the interactions between key cell populations.

### Multiple cancer analysis

**Figure 4.**
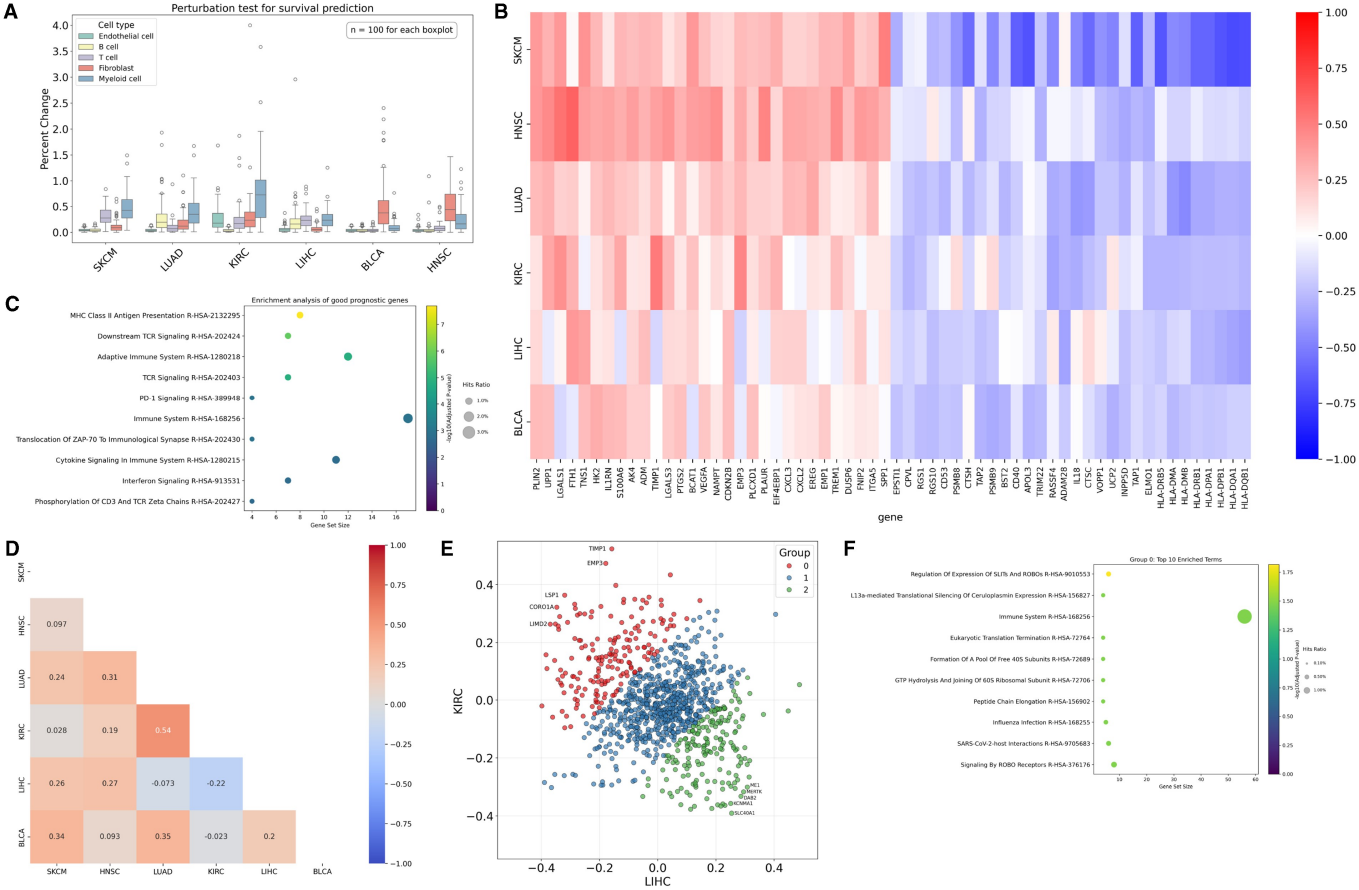
Pan-cancer analysis of stromal cells. (**A**) Permutation test identifying common prognostic cell types across six cancer types. (**B**) Heatmap showing Pearson correlations between myeloid cell gene expression and contributions across six cancer types. The heatmap illustrates the top 30 genes with the highest average correlation and the bottom 30 genes with the lowest average correlation across the cancer types. (**C**) Dot plot showing gene set enrichment analysis results using the top 30 negatively correlated genes with contributions. Dot size represents the proportion of enriched genes, the color indicates statistical significance, and the *x*-axis shows the number of genes in each pathway. (**D**) Cancer similarity based on gene correlations. (**E**) Scatter plot of correlations between gene expression and contributions in liver and kidney cancer myeloid cells. The genes were clustered into three groups by applying a Gaussian mixture model with three components to their projections onto the y=−x line. (**F**) Gene set enrichment analysis results for differentially prognostic genes in liver and kidney cancer myeloid cells.

We performed a scSurv analysis of multiple cancer types. We selected six cancers where the lower bound of the 95% CI exceeded 0.5 in test patients and analyzed the cell populations and genes consistently linked to survival outcomes across these cancers. We first conducted permutation tests on the contribution scores of common cell types across the six cancers, including endothelial cells, fibroblasts, T cells, B cells, and myeloid cells, to determine their relative importance in the hazard function prediction (Fig. 4A). Among the analyzed cell types, myeloid cells had the strongest influence on prediction accuracy. We extracted myeloid cell populations from each cancer type and examined their correlations with the contributions of genes commonly expressed across all cancer types (Fig. 4B). We performed gene set enrichment analysis on the top and bottom genes ranked by mean correlation to elucidate the biological pathways consistently associated with prognosis in myeloid cells across different cancers. Genes associated with a good prognosis were enriched in antigen presentation-related terms (Fig. 4C), consistent with the known role of antigen presentation in antitumor immunity (Jhunjhunwala *et al.* 2021).

Next, we calculated the similarities between the cancer types based on their correlations (Fig. 4D). Notably, myeloid cells from hepatocellular carcinoma and renal cell carcinoma showed opposite correlation patterns. Analysis of differentially correlated genes (Fig. 4E) revealed enrichment of the Slit/Robo signaling pathways (Fig. 4F). These findings suggest that these pathways differentially affect survival outcomes in hepatocellular and renal carcinomas through mechanisms mediated by myeloid cells.

### Application to other clinical outcomes in a COVID-19 cohort

**Figure 5.**
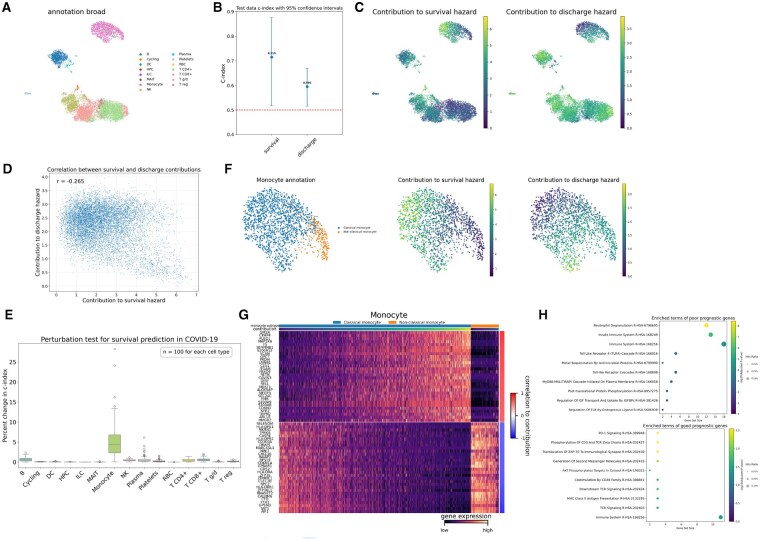
COVID-19 PBMC analysis. (**A**) UMAP visualization of cell type annotations in COVID-19 dataset. (**B**) 95% confidence intervals of scSurv c-indices for survival and discharge in test patients. (**C**) UMAP visualization of single-cell contributions to survival and discharge hazard. Higher values indicate adverse prognostic impact. (**D**) Scatter plot of single-cell contributions for both outcomes. Cellular contributions to survival hazard exhibited an inverse correlation with discharge hazard. (**E**) Permutation test identifying prognostically important clusters in COVID-19. (**F**) UMAP visualization of monocyte annotations and their hazard contributions to both outcomes. Classical monocytes show high contributions to survival hazard but low contributions to discharge hazard. (**G**) Heatmap showing reconstructed expression of genes correlated with estimated contributions to survival hazard. Top 30 positively and negatively correlated genes are displayed. The estimated single-cell contributions are shown in the upper panel. (**H**) Dot plot showing gene set enrichment analysis results using the top 30 positively and negatively correlated genes with contributions to survival hazard. Dot size represents the proportion of enriched genes, the color indicates statistical significance, and the x-axis shows the number of genes in each pathway.

We applied scSurv to hospitalized COVID-19 patients to demonstrate the applicability of our method to acute immune responses beyond cancer and to clinical outcomes beyond survival. Bulk RNA-sequencing data of peripheral blood mononuclear cells (PBMCs) and the corresponding clinical information were obtained from the IMPACC cohort (IMPACC Manuscript Writing Team and IMPACC Network Steering Committee 2021). Single-cell RNA sequencing data from PBMCs of 12 patients in Yoshida et al. (Yoshida *et al.* 2022) were used as a reference dataset (Fig. 5A). We applied scSurv to two distinct clinical outcomes: survival and discharge. scSurv provided robust predictions for both endpoints (Fig. 5B). Here, we found that monocytes exhibited a large contribution to survival hazard (Fig. 5C). Cellular contributions to survival hazard exhibited an inverse correlation with discharge hazard (Fig. 5D), reflecting the clinical relationship between shortened survival and extended hospitalization in severe disease. Permutation testing identified monocytes as the key cellular population in outcome prediction (Fig. 5E). These findings, including the large contribution of monocytes to the survival hazard and their importance identified through permutation testing, align with previous studies demonstrating the importance of monocytes in COVID-19 severity (Knoll *et al.* 2021, Vanderbeke *et al.* 2021). Based on these observations, we focused our subsequent analyses on this subset.

The distribution of contributions between classical and non-classical monocyte subtypes aligned with previous findings on the role of classical monocytes in COVID-19 severity (Vanderbeke *et al.* 2021) (Fig. 5F). The top 30 genes positively correlated with monocyte contributions included S100A12, S100A9, and S100A8, while the top 30 genes negatively correlated with monocyte contributions included HLA-DRA, HLA-DRB1, HLA-DPA1, and HLA-DPB1 (Fig. 5G). High expression of S100A proteins and low expression of HLA-DR proteins in monocytes have been identified as hallmarks of progressive disease (Unterman *et al.* 2022). These gene expression patterns were previously linked to disease severity in an analysis of scRNA-seq data from 66 PBMC samples (Knoll *et al.* 2024). While collecting scRNA-seq data from a large number of individuals is cost-prohibitive, scSurv enables single-cell level analyses by integrating bulk RNA-seq data with a smaller scRNA-seq reference dataset. Our method provides a cost-effective solution for high-resolution profiling. Gene set enrichment analysis further revealed that genes positively correlated with monocyte contributions were enriched in neutrophil degranulation pathway and innate immune system pathway, aligning with reports linking activation of innate immune cells to poor COVID-19 prognosis (Fig. 5H) (Meizlish *et al.* 2021, Sievers *et al.* 2024). Conversely, genes negatively correlated with monocyte contributions were enriched in pathways related to TCR signaling and antigen presentation, supporting findings that impaired adaptive immune responses and antigen presentation are associated with disease severity (Moderbacher *et al.* 2020, Notarbartolo *et al.* 2021). These findings highlight scSurv’s broader applicability beyond cancer and its utility in analyzing time-to-event data beyond survival time.

## Discussion

We present the first methodology to quantify individual cells’ contributions to clinical outcomes. This method allows the investigation of cells linked to patient outcomes using existing cohorts such as TCGA, enabling novel insights into cellular heterogeneity at unprecedented resolution. scSurv does not rely on cell clustering, and its estimations are unaffected by cell labels, providing robust and unbiased results. Additionally, by utilizing the framework of conditional variational autoencoders for batch effect removal, we can effectively integrate scRNA-seq data from multiple patients to use as a reference.

Beyond its biological insights and practical utility, scSurv holds significance as an extension of Cox proportional hazards modeling. While notable deep learning-based Cox models such as DeepSurv (Katzman *et al.* 2018) and Cox-nnet (Ching *et al.* 2018) have enabled more accurate survival predictions, their non-linear nature presents challenges for interpretability. Although methods combining deep learning with linear predictors, such as Cox-PASNet (Hao *et al.* 2019) and PAGE-Net (Hao *et al.* 2020), have been developed, these approaches are not designed to address gene expression and heterogeneity at the individual cell level. scSurv extends the Cox proportional hazards model by incorporating both the estimated proportions of single cells in bulk RNA-seq samples and the contributions of individual cells to clinical outcomes, both derived from latent cell states obtained through a VAE. By leveraging these latent variables, this extended model enables precise survival analysis at the single-cell level and effectively scales to analyze contributions across large numbers of cells.

However, we recognize that scSurv has certain limitations. Similar to existing deconvolution methods, our method cannot assign proportions or contributions to cells that are not included in the reference. Therefore, it is essential to select reference datasets that comprehensively represent the cell populations found in bulk RNA-seq data. Additionally, the method requires a sufficient number of patients (over 300) and cannot be applied to cancers with rare death events. Furthermore, in certain cancer types, bulk RNA-seq data may contain insufficient predictive information, making accurate hazard function estimation difficult. Addressing these constraints remains a priority for future methodological development.

Despite these limitations, scSurv is a novel method to quantitatively evaluate individual cells’ effects on clinical outcomes, enable identification of clinically relevant cell populations and genes, and generate new insights through integration with spatial transcriptomics. This method introduces an innovative concept of cellular heterogeneity based on contributions to clinical outcomes.

In summary, scSurv represents a significant advancement in single-cell analysis methodology, bridging the gap between cellular heterogeneity and clinical outcomes. We anticipate that scSurv will become an essential tool for researchers investigating the cellular basis of disease progression and treatment response, ultimately contributing to the development of more effective, personalized therapeutic strategies.

## Supplementary Material

btaf671_Supplementary_Data

## Data Availability

The implementation of scSurv is available on GitHub (https://github.com/3254c/scSurv) and Zenodo (https://doi.org/10.5281/zenodo.17793054). The single-cell RNA sequencing datasets used in this study are available from the Gene Expression Omnibus (GEO) under accession numbers GSE129845 (kidney cancer)(Li *et al.* 2022b), GSE115978 (melanoma)(Jerby-Arnon *et al.* 2018), GSE129845 (bladder cancer)(Yu *et al.* 2019), GSE131907 (lung adenocarcinoma)(Kim *et al.* 2020), GSE176078 (breast cancer)(Wu *et al.* 2021), GSE132465 and GSE144735 (colorectal cancer)(Lee *et al.* 2020), GSE164690 (head and neck cancer)(Kürten *et al.* 2021), GSE149614 (hepatocellular carcinoma)(Lu *et al.* 2022), and GSE183904 (gastric cancer)(Kumar *et al.* 2022); from ArrayExpress under accession numbers E-MTAB-11948 (cervical cancer)(Li *et al.* 2022a) and E-MTAB-8107 (lung squamous cell carcinoma)(Qian *et al.* 2020); from Cell x Gene Explorer (Collection ID: 4796c91c-9d8f-4692-be43-347b1727f9d8) for ovarian cancer(Vázquez-García *et al.* 2022); and from COVID-19 Cell Atlas (https://covid19cellatlas.org) for COVID-19 PBMCs. Bulk RNA sequencing data and clinical information from TCGA are available from the Genomic Data Commons (GDC) Data Portal (https://portal.gdc.cancer.gov/). The bulk RNA-seq and clinical outcome data from the IMPACC cohort used in this study are accessible via the ImmPort database (https://www.immport.org) with a data access request.
