# Supplementary material for: scSurv: a deep generative model for single-cell survival analysis
Source: Bioinformatics. 2025 Dec 22;42(1):btaf671. doi: 10.1093/bioinformatics/btaf671 (PMC12797213; doi:10.1093/bioinformatics/btaf671)
Supplement: btaf671_Supplementary_Data [file btaf671_supplementary_data.pdf]

scSurv: a deep generative model for single-cell survival analysis

Supplementary Materials

Notes
- Shift invariance of the breslow/efron loss functions
- Model training
- Simulation of deconvolution and survival prediction
- Scalability simulation for scSurv
- Systematic simulation of cell number, bulk number, and noise level
- scRNA-seq data preprocessing
- TCGA bulk data preprocessing
- COVID-19 PBMC bulk data preprocessing
- Generalization performance of scSurv across 12 TCGA cancers
- Spatial transcriptome preprocessing
- Gene set enrichment analysis
- Cell-cell communication analysis
- Multiple cancer analysis

Figures
-Graphical Abstract
-S1
-S2
-S3
-S4

Tables
-S1
-S2

References

Supplementary Notes

Shift invariance of the breslow/efron loss functions

Using the relation $\sum_{c=1}^{C} m_{\theta_{bulk}}(\boldsymbol{z_c})_b = 1$, we obtain:

$$\sum_{c=1}^{C} (\beta_{\theta'}(z_c) - \varepsilon) m_{\theta_{bulk}}(z_c)_b$$

$$= \eta_b - \varepsilon \sum_{c=1}^{C} m_{\theta_{bulk}}(z_c)_b$$

$$= \eta_b - \varepsilon$$

From this fact, when η is translated to $\eta - \varepsilon$ in the equation $\mathcal{L}_{\mathrm{breslow}}$, we get that the loss function is invariant as following.

$$\sum_{b:E_=1} \left((\eta_b - \varepsilon) - \log \sum_{i:T_i \geq T_b} \exp(\eta_i - \varepsilon) \right)$$

$$= \sum_{b:E_b=1} \left(\eta_b - \varepsilon - \log \left(\exp(-\varepsilon) \sum_{i:T_i \geq T_b} \exp(\eta_i) \right) \right)$$

$$= \sum_{b:E_b=1} \left(\eta_b - \varepsilon + \varepsilon - \log \sum_{i:T_i \geq T_b} \exp(\eta_i) \right)$$

$$= \mathcal{L}_{breslow}(\theta')$$

As a same manner, $\mathcal{L}_{\mathrm{efron}}$ is invariant as following.

$$\sum_{b:E_b=1} \left\{ (\eta_b - \varepsilon) - \log \left(\sum_{i:T_i \geq T_b} \exp(\eta_i - \varepsilon) - \frac{r_b - 1}{d_b} \sum_{l:T_l = T_b, E_l = 1} \exp(\eta_l - \varepsilon) \right) \right\}$$

$$= \sum_{b:E_b=1} \left\{ \eta_b - \varepsilon - \log \left(\exp(-\varepsilon) \left(\sum_{i:T_i \geq T_b} \exp(\eta_i) - \frac{r_b - 1}{d_b} \sum_{l:T_l = T_b, E_l = 1} \exp(\eta_l) \right) \right) \right\}$$

$$= \sum_{b:E_b=1} \left\{ \eta_b - \varepsilon + \varepsilon - \log \left(\sum_{i:T_i \geq T_b} \exp(\eta_i) - \frac{r_b - 1}{d_b} \sum_{l:T_l = T_b, E_l = 1} \exp(\eta_l) \right) \right\}$$

$$= \mathcal{L}_{efron}(\theta')$$

Therefore, we used $\beta_{\theta'}(z_c)$ only for relative comparisons between cells. In downstream analyses, we transformed $\beta_{\theta'}(z_c)$ by setting $\varepsilon = \min(\beta_{\theta'}(z_c))$ to ensure that the minimum value equals zero.

Model training

The VAE was trained on 85% of the scRNA-seq data, with 10% allocated for validation and 5% for testing. The training was conducted using mini-batches of 1,000 cells, and the process was terminated when the validation loss averaged over 10 epochs failed to improve for 10 consecutive epochs. The model was optimized using AdamW with a learning rate of 0.01.

After training the VAE and fixing its parameters, the deconvolution model was trained using the same data splitting and training procedures. When the deconvolution model converged, the parameters were fixed.

For the contribution estimation, the validation and test datasets of the cells were the same as in the previous steps, but the mini-batch size for the training data was set to 500 cells. Bulk RNA-seq data were randomly split into 60% training, 20% validation, and 20% test sets to ensure that the proportion of patients with events was approximately balanced across these subsets. For each split (training, validation, and test), the c-index was computed using only the cells and bulk RNA-seq samples contained in that split. The training was terminated when the c-index, computed from the hazard estimates on both the cell and patient validation sets and averaged over 10 epochs, showed no improvement over 10 consecutive epochs. For this step, the AdamW optimizer was used, with a learning rate of 0.0001.

The typical wall times and maximum GPU and CPU usage for each dataset are provided in Table S2. These are the results for scSurv run on a single NVIDIA RTX3090.

Simulation of deconvolution and survival prediction

Pseudo-bulk samples were created using the breast cancer scRNA-seq data from Wu et al [1]. We first selected scRNA-seq data from the patient with the highest cell count and randomly split it in half. We used one half to create pseudobulk samples and the other half as a reference. A total of 300 pseudo-bulk samples were generated in this process. We utilized the pre-assigned 'celltype_minor' annotation as cluster labels. The selection probability for each cluster was generated from a Dirichlet distribution with $\alpha = 1$ to ensure uniform selection across cluster labels. The number of cells extracted from each cluster was generated from a multinomial distribution by using these selection probabilities. The total cell count for each pseudobulk was sampled from a discrete uniform distribution $U\{1000, 10000\}$.

Following Austin's methodology [2], we simulated the survival time T_b for each bulk sample:

$$T_b = -\frac{\log(u_b)}{h(t)_b}$$

$$h(t)_b = h_0(t) \exp\left(\sum_{n=1}^{N} \beta_n P_n^b\right)$$

$$h_0(t) = \lambda$$

where $u_b \sim U(0,1)$ (standard uniform distribution), $\lambda = 0.001$, N is the number of 'celltype_minor' clusters, $\beta \sim \mathcal{N}(0, 5^2 I)$, and P represents the cell proportion of each cluster in each bulk sample. To evaluate the accuracy of regression coefficient estimation, we generated 100 datasets using the same 300 pseudo-bulk samples, each with different survival time settings obtained by varying the β values. All samples were set to be uncensored.

Scalability simulation for scSurv

In Fig. S3, we performed simulations to examine the scalability of scSurv with respect to the number of input cells. We used the ovarian cancer single cell RNA sequencing dataset in Vázquez García et al. We randomly sampled up to 500,000 cells as the single cell reference and constructed pseudo bulk data from the remaining cells. Using the 'cluster_label' annotation, we created the pseudo bulk and assigned survival times in the same manner as in the breast cancer simulation. For this simulation, wall time and CPU/GPU memory measurements were performed using an NVIDIA RTX6000 Ada.

Systematic simulation of cell number, bulk number, and noise level

In Fig. S4, we performed a systematic simulation over cell count, bulk size, and noise level in the cell labels. As in the section titled 'Simulation of deconvolution and survival prediction', we used the breast cancer scRNA-seq data of Wu et al[1]. For each number of cells, we randomly sampled from all cells to construct the single cell reference, and we created the pseudo bulk from the remaining cells. For the reference single cell data in the existing methods, we used either the 'celltype_minor' labels or the labels obtained by subclustering the 'celltype_major' labels. Because the single cell data include multiple patients, before running Leiden clustering we obtained PCA after batch correction using the 'scanpy.external.pp.harmony_integrate' and computed the neighbor graph from that PCA. The same neighbor graph was used to compute the UMAP shown in Fig. S4A to S4D. Leiden clustering was performed with the function 'scanpy.tl.leiden' with resolution values of 0.15, 0.30, and 0.50.

scRNA-seq data preprocessing

All transcriptome count data were processed using the Scanpy Python package. For cancer reference scRNA-seq data exceeding 10,000 cells, we randomly sampled 10,000 cells. For COVID-19 reference scRNA-seq data [3], cells annotated as 'COVID_status: COVID-19' and 'Group: Adult' were included to ensure consistency with the bulk RNA-seq data. From these filtered cells, 10,000 cells were randomly sampled. Genes expressed in less than 1% of all cells were excluded. This gene filtering criterion was similarly applied to the bulk and spatial transcriptome datasets. We retained genes common to both scRNA-seq and bulk RNA-seq datasets. We selected the top 5,000 highly variable genes in bulk RNA-seq using `scanpy.pp.highly_variable_genes` and used them as inputs for scSurv.

TCGA bulk data preprocessing

Clinical information and bulk RNA-seq data were obtained from the Genomic Data Commons (GDC) Data Portal. For patients with multiple bulk RNA-seq samples, we prioritized biopsies from primary tumors, followed by metastatic tumors if the primary tumors were unavailable. When two biopsies were obtained from the same site, the biopsy with the highest total count was selected. Samples lacking both death time and the last contact time were excluded. Following Liu et al. [4], we obtained sample procurement time from the 'days_to_sample_procurement' field. For ovarian cancer data, for which most sample procurement times were missing, we set the procurement time equal to the diagnosis time. For other cancers, we excluded data with missing sample procurement dates and calculated the survival time from the procurement time as time zero.

COVID-19 PBMC bulk data preprocessing

Clinical information and bulk RNA-seq data were obtained from the IMPACC cohort [5]. This cohort includes 1164 hospitalized COVID-19 patients, aiming to investigate immune responses, disease progression, and potential biomarkers. We used bulk RNA-seq data from PBMCs. For patients with multiple samples, we selected samples collected when the patient's respiratory status was the most severe. We excluded samples that were collected after discharge or death, along with those missing survival or discharge data. After applying these exclusion criteria, 379 patients with available PBMC bulk RNA-seq data were used as input for scSurv. In this study, we focused on survival and discharge as the outcomes because they are common endpoints in time-to-event data analysis and are relevant for understanding disease progression and recovery in hospitalized patients.

Generalization performance of scSurv across 12 TCGA cancers

We applied scSurv to 12 cancer types with over 300 patients and non-rare death events. We performed 100 different training-validation-test splits using different random seeds, applying scSurv once for each seed. To quantitatively evaluate prediction accuracy, we calculated the c-index for the training, validation, and test data using the `lifelines.utils.concordance_index` function from the lifelines library. To report insights not captured by the c-index alone, we also calculated the Integrated Brier Score (IBS). This was calculated for 100 uniform time points within the minimum and 90th percentile survival times in the test set, as in Wissel et al [6]. For comparison, we performed a regression on the hazard function using dimensionally reduced bulk RNA-seq counts. Regression was performed using the `CoxPHFitter` function from the lifelines library. We set penalizer=0.01 to prevent convergence errors. Using the same training data as scSurv, we performed a hazard function regression and calculated the c-indices and IBS of the test data.

Spatial transcriptome preprocessing

We obtained Visium data for kidney cancer from Li et al.'s [7] public dataset. Following their criteria, we excluded Visium spots with counts below 2,000 or above 35,000, spots with fewer than 500 genes, and spots with mitochondrial gene percentages exceeding 20%. In addition, we removed two low-quality slides (6800STDY12499504 and 6800STDY12499505) with < 500 spots. We retained the genes that were commonly detected across scRNA-seq, bulk RNA-seq, and spatial transcriptome data. We selected the top 1000 highly variable genes in bulk RNA-seq using `scanpy.pp.highly_variable_genes` and used them as inputs for scSurv.

The cell proportions for each spot were estimated using the same method that was used for bulk deconvolution in scSurv. Using the estimated survival time contributions $\beta_{\theta'}(\boldsymbol{z_c})$ and cell proportions in Visium spots $m_{\theta^{spot}}(\boldsymbol{z_c})$, we defined the hazard function for each spot as:

$$h(t)_s = h_0^{spot}(t)\exp(\eta_s)$$

$$\eta_s = \sum_{c=1}^{C} \beta_{\theta'}(z_c) m_{\theta^{spot}}(z_c)_s$$

where s denotes the spot, t is time, and $h_0^{spot}(t)$ is the baseline hazard function. For visualization, we divided each spot's hazard by the mean hazard across spots to eliminate the baseline hazard function.

Permutation test

For permutation tests, we randomly reassigned contributions $\beta_{\theta'}(\boldsymbol{z_c})$ to the hazard function among cells within each cell type cluster. We then recalculated c-indices using all patients to determine how the permutation of each cell type affected the c-index. Cell types contributing to large c-index changes were considered necessary for prognosis prediction.

Gene set enrichment analysis

We performed gene set enrichment analysis using GSEApy [8]. We used the 'Reactome_2022' database for the gene set. In the analyses of melanoma, renal cell carcinoma, and COVID-19, we used the input genes for scSurv as the background. For the pan-cancer analysis, the overlapping genes from scSurv inputs across the six cancers were used as the background.

Cell-cell communication analysis

Following the methodology of Kojima et al. [9], we extracted the activated ligand-receptor relationships between identified T cells and non-T cells in the kidney cancer dataset. Using spatial transcriptome data, we first extracted cell pairs that co-localized in the same spots at rates twice as high as expected from random distribution. We then extracted pairs consisting of 'CD8+T_cycling-G2/M' cluster cells and other cell types from the co-localized pairs. Using genes expressed in these pairs showing expression in the top 5% percentile, we investigated activated cell-cell communication using NicheNet [10].

Multiple cancer analysis

We analyzed six cancers showing c-indices above 0.5 in test data: melanoma, lung adenocarcinoma, renal cell carcinoma, hepatocellular carcinoma, head and neck cancer, and bladder cancer. Cell type annotations were available from the original papers for all cancers except bladder cancer, which we annotated using the marker genes from Yu et al [11]. In hepatocellular carcinoma, we sub-clustered the 'T/NK' cluster into T cell and NK cell clusters using the NCR1 gene.

Supplementary Figures

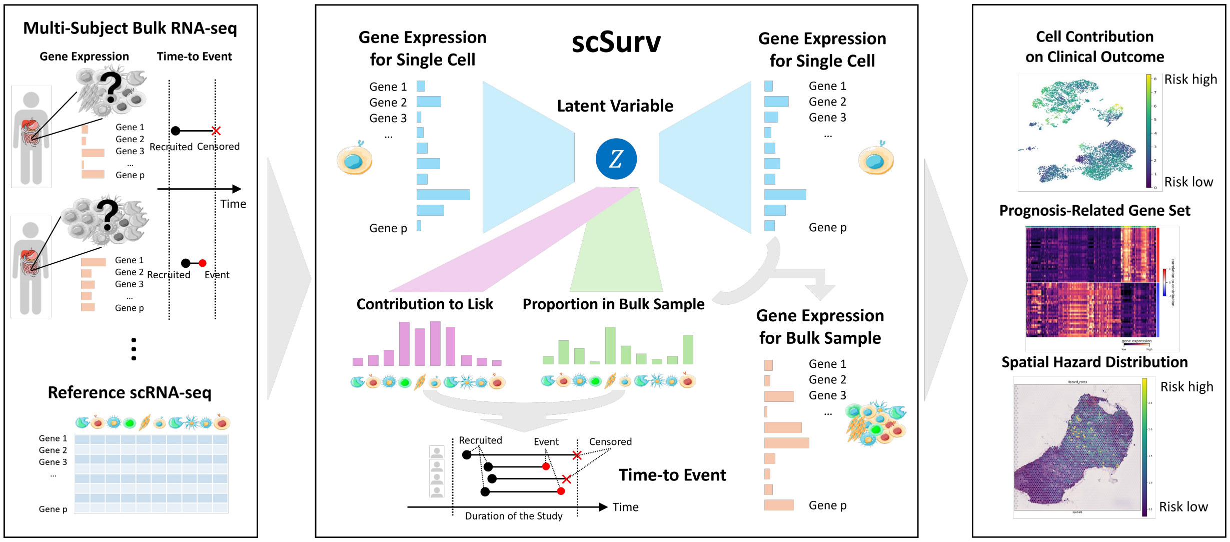

Graphical Abstract

Overview of scSurv, a deep generative model for single-cell survival analysis. This method deconvolutes bulk RNA-seq data into each single cell using VAE, followed by survival analysis with an extended Cox proportional hazards model. The framework enables single-cell level prognostic analysis, identification of outcome-associated genes, and spatial hazard mapping.

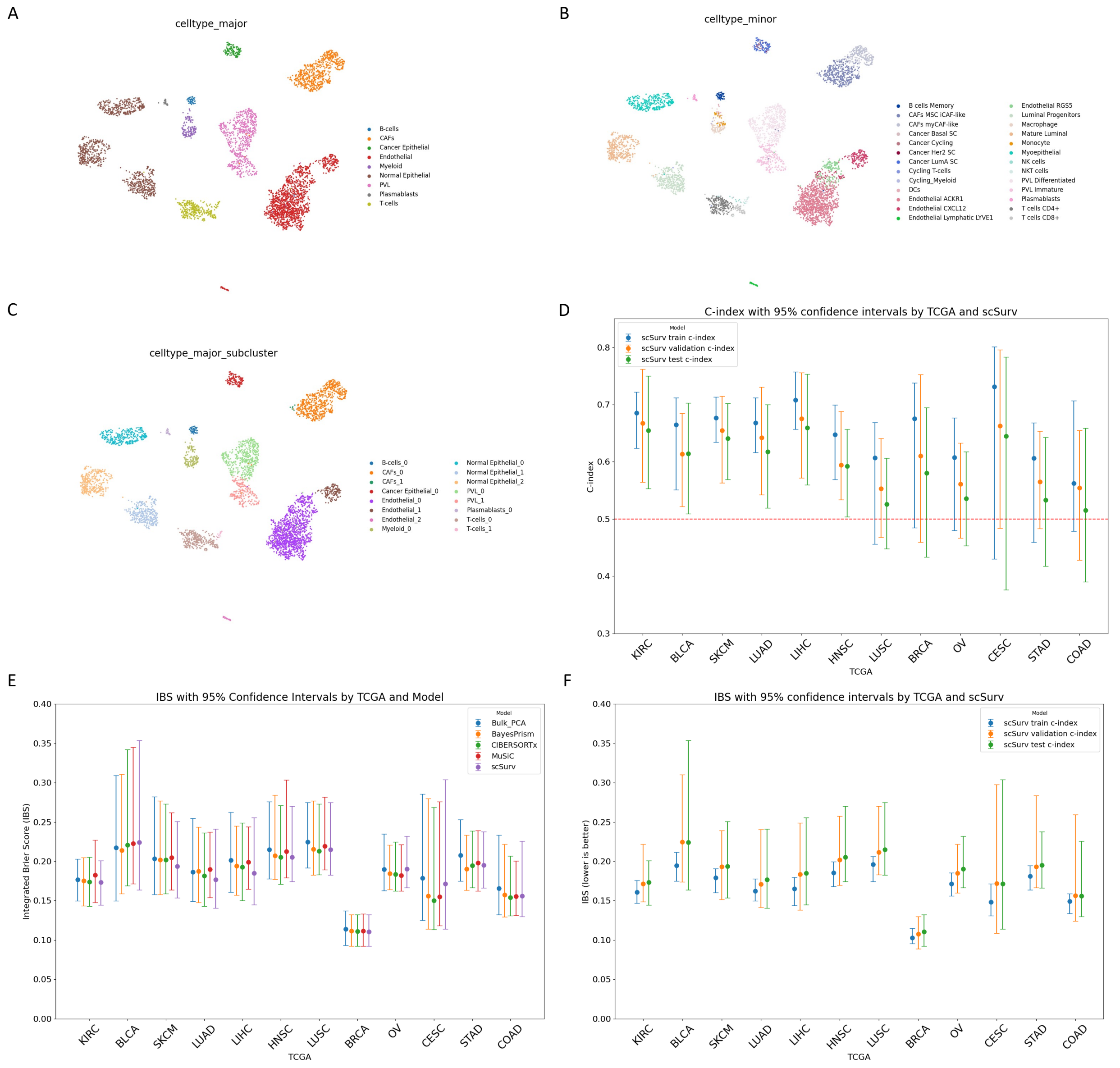

Fig. S1. Overview of simulation data and scSurv performance.

(**A**) UMAP visualization of 'celltype_major' clusters. (**B**) UMAP visualization of 'celltype_minor' clusters used for generating pseudo-bulk samples in simulation. (**C**) UMAP visualization of 'celltype_major' subclusters, generated using sc.tl.leiden() with resolution=0.10. These cluster labels were used as alternative annotations for BayesPrism, CIBERSORTx, and MuSiC in simulation. (**D**) scSurv performance is measured by c-index across training, validation, and testing patients for 12 cancer types. (**E**) The performance of scSurv, the combination of the existing methods for bulk deconvolution with the Cox proportional hazards model, and the combination of bulk PCA with the hazard model were evaluated across 12 TCGA cancer types using IBS. (**F**) scSurv performance is measured by IBS across training, validation, and testing patients for 12 cancer types.

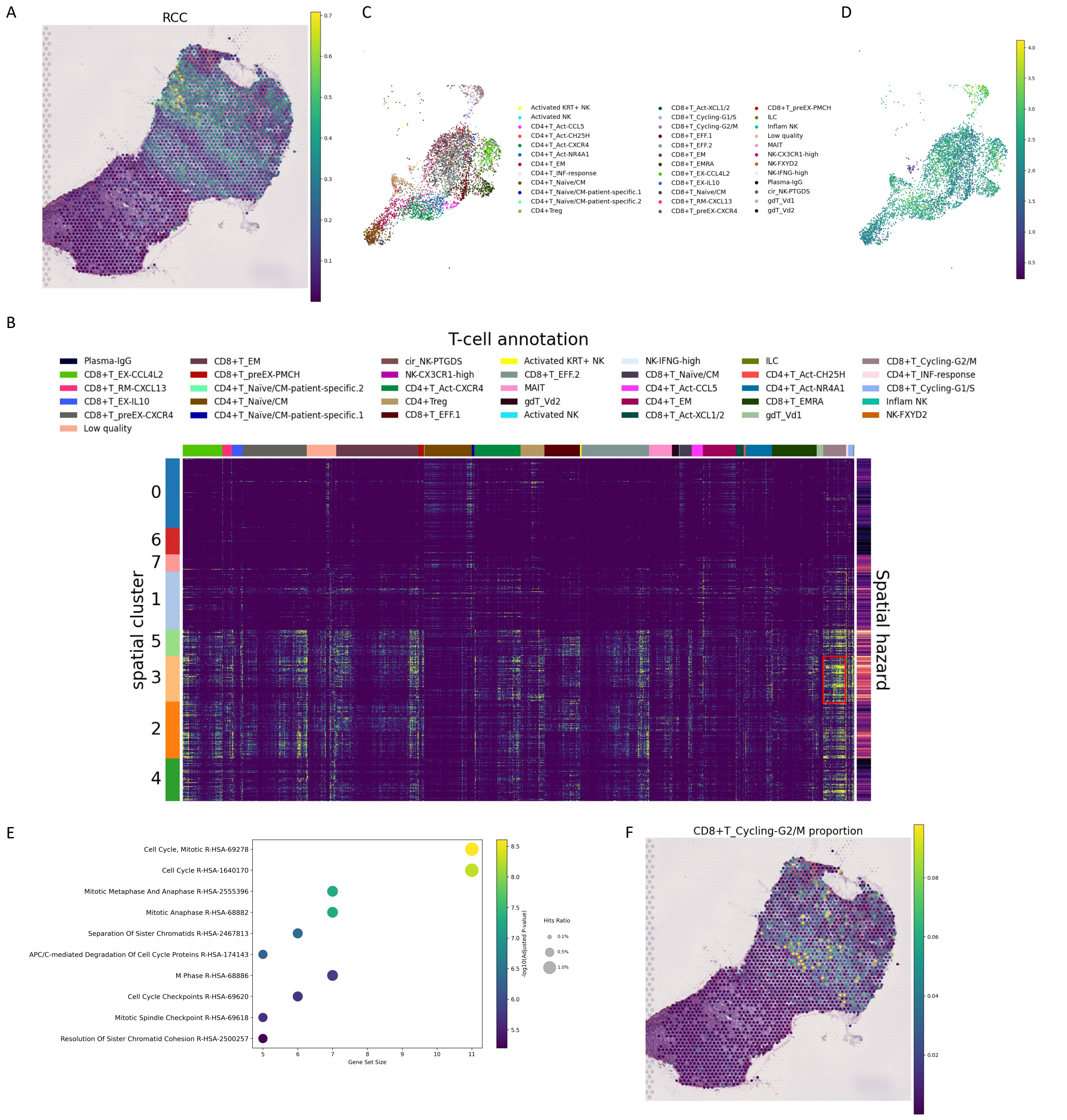

Fig. S2. Integration of scSurv with spatial transcriptomics identifies proliferative CD8+ T cells driving hazard in RCC.

(**A**) The proportion of cancer cells in each spot estimated by scSurv. (**B**) Heatmap of single-cell contributions adjusted for cell proportions in T cells. 'CD8+T_cycling-G2/M' cluster is specific to cluster 3. (**C**) UMAP visualization of T cell subtypes. (**D**) UMAP visualization of contributions in T cells. (**E**) Gene set enrichment analysis results for differentially expressed genes in the 'CD8+T_cycling-G2/M' cluster. (**F**) Spatial distribution of 'CD8+T_cycling-G2/M' cluster proportion.

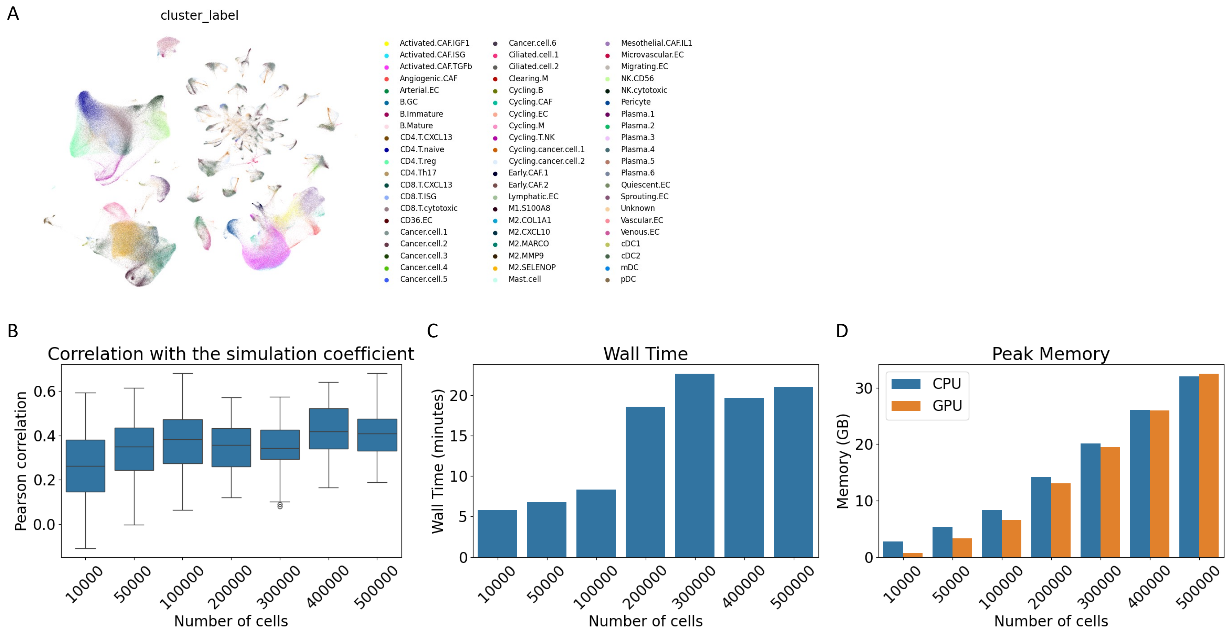

Fig.S3. Scalability simulation for scSurv.
(A) UMAP visualization of 'cluster_label' clusters of ovarian cancer scRNA-seq dataset. **(B)** Pearson correlation between the set regression coefficients and the estimates from scSurv. For datasets with different numbers of cells, we assigned 100 distinct sets of regression coefficients and survival times and applied scSurv once to each. **(C)** Run time of scSurv on simulation data with different numbers of cells. **(D)** Peak CPU and GPU memory during the scalability simulation.

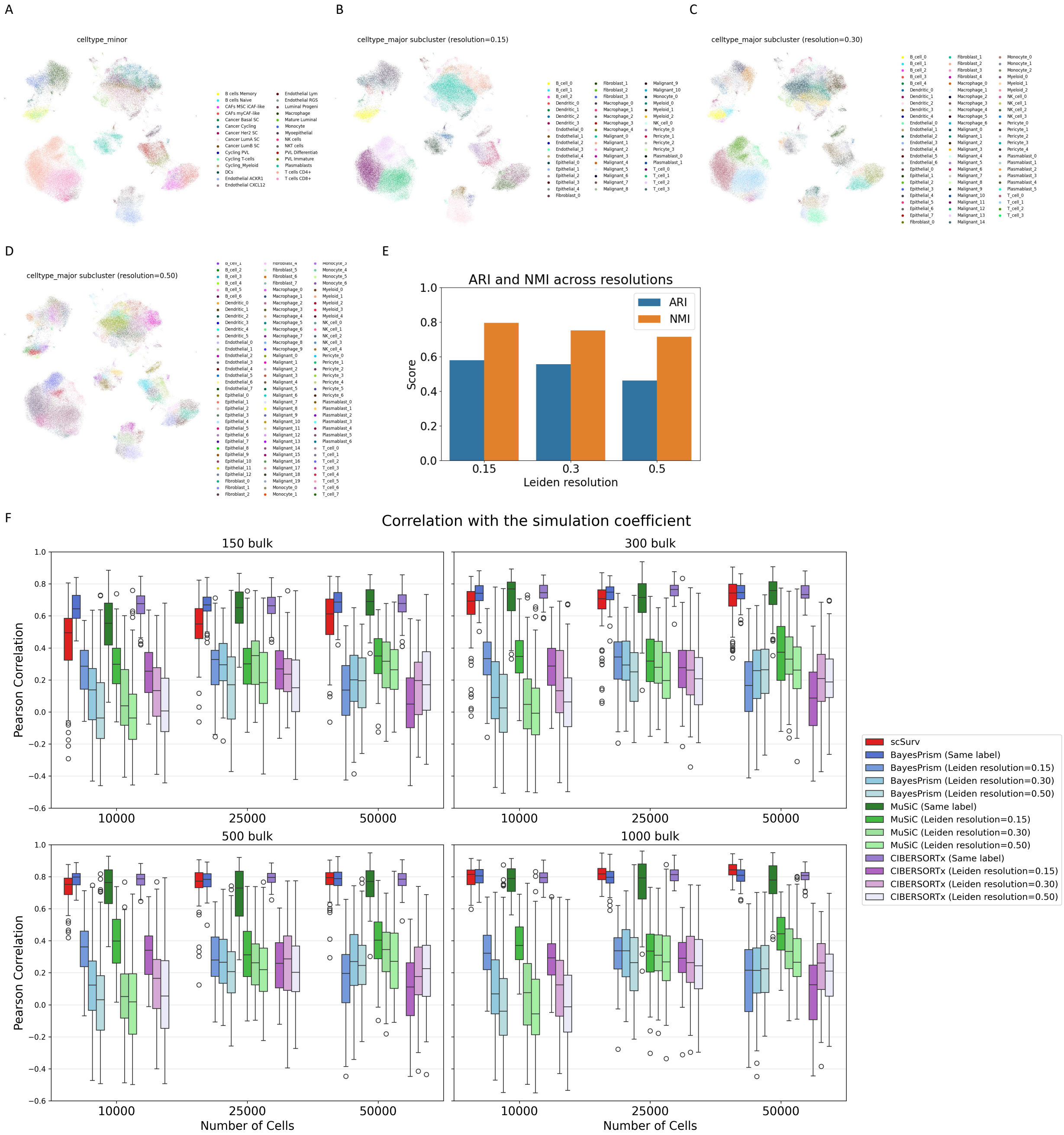

Fig.S4. Systematic simulation of cell number, bulk number, and noise level.
(A) UMAP visualization of celltype_minor' clusters of breast cancer scRNA-seq dataset in this systematic simulation. **(B)** UMAP visualization of 'celltype_major' subclusters, generated using sc.tl.leiden() with resolution=0.15. **(C)** UMAP visualization of 'celltype_major' subclusters, generated using sc.tl.leiden() with resolution=0.30. **(D)** UMAP visualization of 'celltype_major' subclusters, generated using sc.tl.leiden() with resolution=0.50. These cluster labels were used as alternative annotations for BayesPrism, CIBERSORTx, and MuSiC in simulation. **(E)** ARI and NMI scores measuring the concordance between labels derived from Leiden clustering and the 'celltype_major' clusters used to create the pseudo bulk. **(F)** Systematic simulation across different numbers of cells, sizes of bulk samples, and levels of label noise for existing methods.

Table S1: A summary of the architecture, optimization settings, and hyperparameters

Category	Parameter	Setting
General	Hyperparameter search	Fixed a priori
	Dropout	0
	Activation function	GeLU
	Batch normalization	Yes
	Hidden dimensionality	100
	Train/validation/test split (cell)	0.85/0.1/0.05
	Train/validation/test split (patient)	0.6/0.2/0.2
VAE	Mini batch size	1000
	Latent dimensionality	20
	Encoder hidden layers widths	1
	Decoder hidden layers widths	1
	Assuming distribution	Poisson
Deconvolution network	Mini batch size	1000
	Hidden layer widths	1
	Assuming distribution	Negative Binomial
	Dispersion parameter setting	per gene
Survival prediction network	Mini batch Size	500
	Hidden layer widths	1
AdamW hyperparameters	Learning rate (VAE, Deconvolution)	0.01
	Learning rate (Survival prediction)	0.0001
	Weight-decay	0.01
	β -coefficients	(0.9, 0.999)
	Early stopping patience	10

Table S2: The typical wall times and maximum GPU and CPU usage for each dataset

Data	wall-time	CPU memory	GPU memory
SKCM	13m 26s	4.765 GB	0.505 GB
BLCA	20m 54s	4.903 GB	0.681 GB
HNSC	18m 37s	4.828 GB	0.690 GB
STAD	11m 45s	4.776 GB	0.685 GB
LUAD	20m 21s	4.858 GB	0.693 GB
BRCA	14m 19s	5.355 GB	0.732 GB
KIRC	11m 15s	4.251 GB	0.691 GB
OV	18m 10s	4.444 GB	0.687 GB
COAD	24m 47s	4.936 GB	0.690 GB
LIHC	18m 8s	4.921 GB	0.679 GB
LUSC	25m 20s	4.691 GB	0.551 GB
CESC	16m 5s	4.393 GB	0.674 GB
KIRC(spatial,1000genes)	14m 10s	8.590 GB	0.289 GB
COVID(survival)	8m 41s	6.975 GB	0.681 GB
COVID(discharge)	11m 7s	5.381 GB	0.681 GB

Supplementary References

[1] Wu, S. Z., Al-Eryani, G., Roden, D. L., et al. (2021) A single-cell and spatially resolved atlas of human breast cancers. Nature Genetics 53.9, pp. 1334–1347.
[2] Austin, P. C. (2012) Generating survival times to simulate Cox proportional hazards models with time-varying covariates. Statistics in Medicine 31.29, pp. 3946–3958.
[3] Yoshida, M., Worlock, K. B., Huang, N., et al. (2021) Local and systemic responses to SARS-CoV-2 infection in children and adults. Nature 602.7896, pp. 321–327.
[4] Liu, J., Lichtenberg, T., Hoadley, K. A., et al. (2018) An Integrated TCGA Pan-Cancer Clinical Data Resource to Drive High-Quality Survival Outcome Analytics. Cell 173.2, 400–416.e11.
[5] IMPACC Manuscript Writing Team and IMPACC Network Steering Committee (2021) Immunophenotyping assessment in a COVID-19 cohort (IMPACC): A prospective longitudinal study. Science Immunology 6.62, eabf3733.
[6] Wissel, D., Rowson, D., and Boeva, V. (2023) Systematic comparison of multi-omics survival models reveals a widespread lack of noise resistance. Cell Rep Methods 24;3(4):100461.
[7] Li, R., Ferdinand, J. R., Loudon, K. W., et al. (2022) Mapping single-cell transcriptomes in the intra-tumoral and associated territories of kidney cancer. Cancer Cell 40.12, 1583– 1599.e10.
[8] Fang, Z., Liu, X., and Peltz, G. (2023) GSEApy: a comprehensive package for performing gene set enrichment analysis in Python. Bioinformatics 39.1. Published online: 25 November 2022, btac757.
[9] Kojima, Y., Mii, S., Hayashi, S., et al. (2024) Single-cell colocalization analysis using a deep generative model. Cell Systems 15.2, 180–192.e7.
[10] Browaeys, R., Saelens, W., and Saeys, Y. (2020) NicheNet: modeling intercellular communication by linking ligands to target genes. Nature Methods 17.2, pp. 159–162.
[11] Yu, Z., Liao, J., Chen, Y., et al. (2019) Single-Cell Transcriptomic Map of the Human and Mouse Bladders. Journal of the American Society of Nephrology 30.11, pp. 2159–2176.
